# The Tobamoviral Movement Protein: A “Conditioner” to Create a Favorable Environment for Intercellular Spread of Infection

**DOI:** 10.3389/fpls.2020.00959

**Published:** 2020-06-24

**Authors:** Ekaterina V. Sheshukova, Natalia M. Ershova, Kamila A. Kamarova, Yuri L. Dorokhov, Tatiana V. Komarova

**Affiliations:** ^1^Vavilov Institute of General Genetics, Russian Academy of Sciences, Moscow, Russia; ^2^Faculty of Bioengineering and Bioinformatics, Lomonosov Moscow State University, Moscow, Russia; ^3^Belozersky Institute of Physico-Chemical Biology, Lomonosov Moscow State University, Moscow, Russia,

**Keywords:** tobamovirus movement protein, tobacco mosaic virus, plasmodesmata gating, plasmodesmata-associated proteins, plasmodesmal localization signal, β-1,3-glucanase, pectin methylesterase, synaptotagmin A

## Abstract

During their evolution, viruses acquired genes encoding movement protein(s) (MPs) that mediate the intracellular transport of viral genetic material to plasmodesmata (Pd) and initiate the mechanisms leading to the increase in plasmodesmal permeability. Although the current view on the role of the viral MPs was primarily formed through studies on tobacco mosaic virus (TMV), the function of its MP has not been fully elucidated. Given the intercellular movement of MPs independent of genomic viral RNA (vRNA), this characteristic may induce favorable conditions ahead of the infection front for the accelerated movement of the vRNA (*i.e.* the MP plays a role as a “conditioner” of viral intercellular spread). This idea is supported by (a) the synthesis of MP from genomic vRNA early in infection, (b) the Pd opening and the MP transfer to neighboring cells without formation of the viral replication complex (VRC), and (c) the MP-mediated movement of VRCs beyond the primary infected cell. Here, we will consider findings that favor the TMV MP as a “conditioner” of enhanced intercellular virus movement. In addition, we will discuss the mechanism by which TMV MP opens Pd for extraordinary transport of macromolecules. Although there is no evidence showing direct effects of TMV MP on Pd leading to their dilatation, recent findings indicate that MPs exert their influence indirectly by modulating Pd external and structural macromolecules such as callose and Pd-associated proteins. In explaining this phenomenon, we will propose a mechanism for TMV MP functioning as a conditioner for virus movement.

## Introduction

Since viral molecules are too large for passive transport through plasmodesmata (Pd) by diffusion, viral genomes during evolution acquired genes encoding specific proteins that can induce plasmodesmal dilation. This phenomenon was first described through fundamental studies using tobacco mosaic virus (TMV), the first discovered filtrate infectious agent (Ivanowski, 1892; Beijerinck, 1898; Lechevalier, 1972; Komarova et al., 2011; Dorokhov et al., 2018b). TMV spread through the plant begins with penetration into a single cell of the leaf, followed by virion stripping and the synthesis of nonstructural viral proteins in the cell (Wu et al., 1994; Wu and Shaw, 1996; Nelson and van Bel, 1998). This step is necessary for the formation of the nucleoprotein progeny that move within the cell to the Pd and then through the Pd to the neighboring cells (Waigmann et al., 2004; Niehl and Heinlein, 2011; Ueki and Citovsky, 2011). Successful systemic infection of a plant with TMV requires three processes that repeat over time: initial accumulation and formation of transport form in invaded cells, intercellular movement, and systemic transport (Waigmann et al., 2004; Liu and Nelson, 2013; Heinlein, 2015; Ishibashi and Ishikawa, 2016; Dorokhov et al., 2018b). Biologically, the movement of viruses from cell to cell is a prelude to the massive systemic invasion of the entire host plant, which begins when the viral material reaches the vascular system of the host. TMV evolutionarily acquired the ability to quickly reach the phloem for its movement into the upper sink leaves or to roots, dependent on leaf position (Samuel, 1934; Cheng et al., 2000; Liu and Nelson, 2013). A contemporary view of intercellular transport of viruses grew from the concept of a viral “transport protein” (Atabekov and Dorokhov, 1984), “translocation protein” (Leonard and Zaitlin, 1982), or “movement protein” (MP) (Atabekov and Dorokhov, 1984; Deom et al., 1987). The hypothesis that a nonstructural TMV-encoded 30 kDa protein can facilitate viral spread (Atabekov and Morozov, 1979) was soon confirmed by the study of and use of information from the temperature-sensitive cell-to-cell movement mutant Ls1 (Leonard and Zaitlin, 1982; Taliansky et al., 1982; Deom et al., 1987; Meshi et al., 1987). Two mechanisms were initially proposed to explain the function of the MP (Atabekov and Dorokhov, 1984). In the first mechanism, Pd, originally closed to the virus, are modified by MP and become permeable to viral genetic material. Thus, the MP functions to open, or gate, Pd. The second mechanism suggested that MP did not affect Pd but rather stimulated cellular mechanisms to overcome the resistance of plant cells to the virus and allow intercellular spread. Studies of TMV and other viruses reported the basic properties of MP, including the ability to (a) increase plasmodesmal permeability for translocation of viral complexes, (b) localize to and move through Pd, and (c) bind RNA (Tilsner et al., 2014; Waigmann et al., 2004; Ueki and Citovsky, 2014; Heinlein, 2015; Navarro et al., 2019; Reagan and Burch-Smith, 2020).

However, the mechanisms of viral MP function have not been fully elucidated. Of particular interest is the self-movement property of TMV MP. MP can move into Pd, increase their size exclusion limit (SEL) (*i.e.* gate Pd), and travel to neighboring cells (Crawford and Zambryski, 2001; Kotlizky et al., 2001; Burch-Smith and Zambryski, 2010). Also, in accordance with the diffusion model of cell-to-cell spread (Guenoune-Gelbart et al., 2008; Epel, 2009), TMV MP forms a large complex that binds both the ER and viral RNA and can passively diffuse through Pd. However, additionally MP’s ability to move independently from viral RNA (vRNA) can serve as the basis for the mechanism of cell conditioning and the creation of a favorable environment ahead of the infection front for the accelerated movement of genomic vRNA (Kawakami et al., 2004).

Here, we assess findings that support the concept that the MP creates a conducive environment for viral infection by conditioning cells for infection in advance of the viral genomic RNA.

## MP Is Transiently Synthesized in the Early Stages of a Productive Infection

TMV moves rapidly between cells, needing 16–18 h (Nilsson-Tillgren et al., 1969) to reach the vascular tissue for systemic infection of the host. Particular viral proteins should therefore be synthesized rapidly to provide this spread and the MP would be such a candidate. In support of this view, one can cite the results of experiments obtained more than 20 years ago that describe the spread of infection of TMV expressing a fusion protein MP-GFP. Even as MP-GFP fusion protein, the MP retained its independent movement and contributed to the development and expansion of infection, including the necrosis phenotype, by the TMV encoding MP-GFP (Heinlein et al., 1995; Epel et al., 1996; Oparka et al., 1996; Padgett et al., 1996; Boyko et al., 2000). The TMV-based vectors used in these experiments differed significantly in the amount of MP produced, as was shown in protoplasts (Szécsi et al., 1999). However, the size of the infection focus was independent of the amount of synthesized MP-GFP. This finding indicated that low and transient levels of MP-GFP were essential at the leading edge of an expanding focus of infection. Indeed, in support of the latter assumption, TMV MP-GFP could open Pd only at the leading edge of a focus of infection and did not show this ability at the later stages of infection at the focus center even though MP-GFP was still present in those Pd (Oparka et al., 1997).

Experiments studying the growing edge of the infection focus are also interesting because the behavior of MP-GFP was different from the behavior of the recombinant MP produced in *E. coli*, which appeared in cells distant from the injected cell after microinjection (Waigmann et al., 1994). Experiments in which DNA encoding MP-GFP was delivered into cells using low-pressure microprojectile bombardment without injuring the leaf (Crawford and Zambryski, 2000; Crawford and Zambryski, 2001) or MP-2xGFP-encoding plasmid delivered *via* agroinfiltration (Burch-Smith and Zambryski, 2010) demonstrated that TMV MP, similar to the cucumber mosaic virus 3a MP (Itaya et al., 1997) or the tomato spotted wilt virus NSm MP (Huang et al., 2020), showed autonomy from other viral factors, *i.e.*, could independently move to neighboring cells in the absence of viral RNA, but remained relatively close to the initial cell with the introduced MP-encoding DNA. MP-GFP trafficked to an average of eight (Crawford and Zambryski, 2000) or nine (Crawford and Zambryski, 2001) cells adjacent to the bombarded cell. However, the distance of such movement from the transfected cell was noticeably less than that after microinjection with *E. coli*-produced MP (Waigmann et al., 1994), which could be explained by the inability of bacterially synthesized MP to be phosphorylated (Padgett et al., 1996). If we imagine that in the leading edge of expanding TMV infection sites the synthesized MP-GFP moves independently of the viral RNA into neighboring cells, the introduction of incisions as close as 50 µm, *i.e.*, within one epidermal cell diameter (as was performed in the study by Oparka et al., 1997), could accidentally cut off cells already containing MP-GFP that were not fluorescent yet at the time of surgery because immature GFP needs time for proper folding of the fluorophore (Cubitt et al., 1995). The absence of visible fluorescence on the opposite side of the incision 24 h after the incision could be due to the “dissolution” of MP-GFP, *i.e.*, the decrease in its concentration in every next cell where it moves. The same effect of the gradient reduction of the fluorescence intensity from the primary transfected cell to the second and third order of cells was observed, for example, in experiments in which a plasmid encoding MP-GFP was introduced into individual cells, and GFP fluorescence faded with the distance from the plasmid-containing cell (Burch-Smith and Zambryski, 2010). Thus, although studies of the growing focus of infection (Oparka et al., 1997) did not allow authors to conclude that MP-GFP can move into cells of the growing edge of the focus ahead of viral RNA, experiments with the introduction of a leaf incision in front of the edge of the focus did not fully exclude this possibility.

Given the ability of MP to move independently from cell to cell, it can be hypothesized that MP-GFP, ahead of the front of the viral RNA spread, moves to neighboring healthy cells and creates favorable conditions for the development of infection. The idea of cell “conditioning” or “predisposing” (Kawakami et al., 2004) came from studies showing that TMV MP is produced in protoplasts within 5–8 h post infection (hpi) (Watanabe et al., 1984; Más and Beachy, 1999). Real-time monitoring of the spread of infection in tobacco leaves inoculated with transcripts of the TMV-based construct encoding MP-GFP (Padgett et al., 1996; Szécsi et al., 1999) showed that in epidermal cells in the vicinity of the initially infected cell, one can detect viral replication complex (VRC)-like structures as early as 18–20 hpi. Then, after an additional 2–4 h, VRCs can be detected in distant cells (tertiary cells), followed by a repeat of this process. In primary infected cells, the cycle is reduced by approximately 4 h, and when the virus is transported between the 2nd and 3rd cells, it is reduced from 3.5 to 1.7 h (Kawakami et al., 2004). Notably, these calculations were made using a vector encoding fused MP-GFP protein, the synthesis of which is significantly suppressed due to a decrease in the “strength” of the subgenomic (sg) promoter directing MP-GFP sgRNA synthesis (Epel et al., 1996; Padgett et al., 1996; Szécsi et al., 1999).

**Figure 1:**
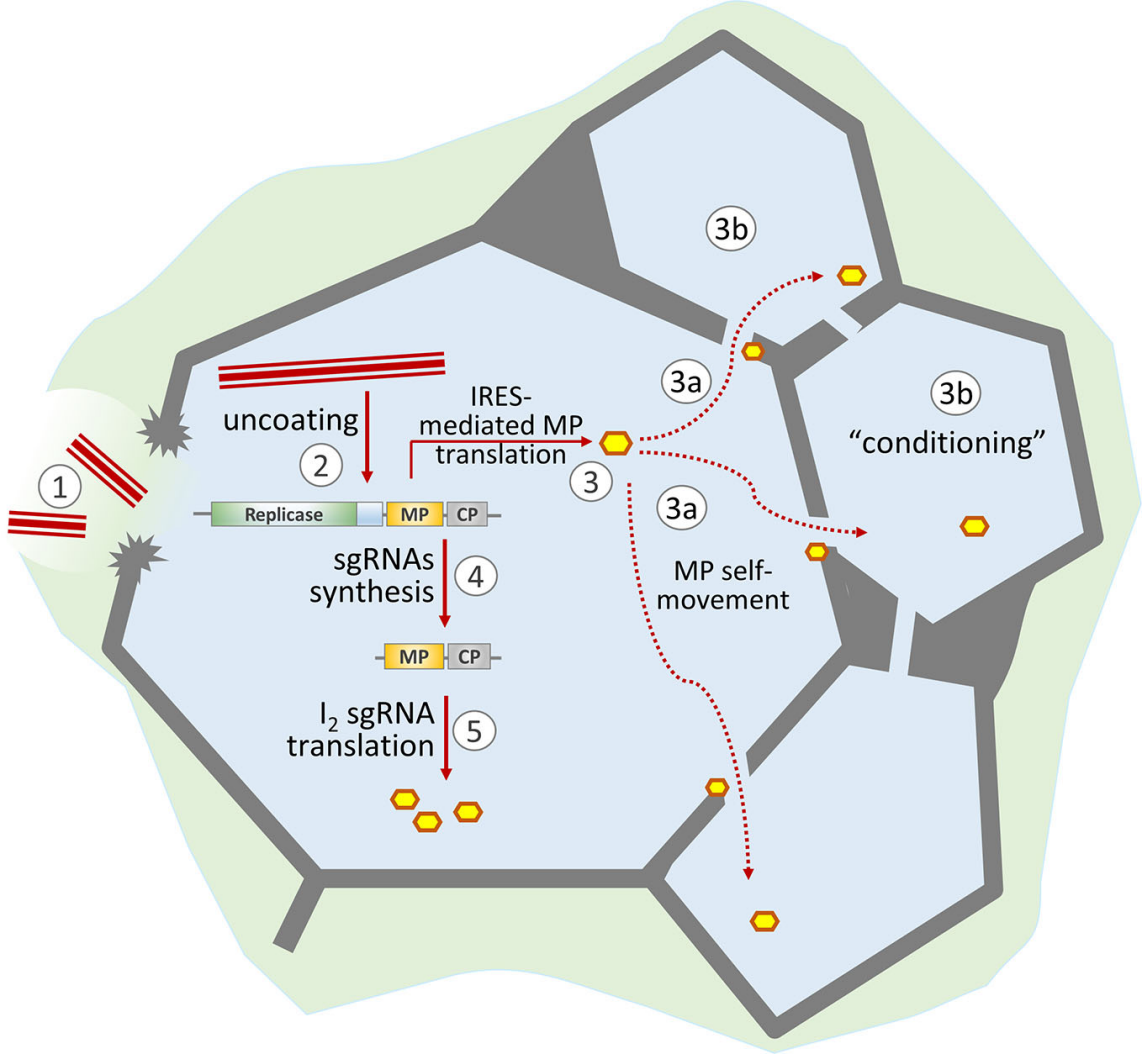
Schematic representation of the role of MP in the development of TMV infection. TMV virions enter the cell through damage to the cell wall (1) and start uncoating (2). The released viral genomic RNA is translated both in a 5′-end-dependent manner (resulting in replicase formation) and *via* the internal ribosome entry site (IRES) to produce MP (3). Due to its self-movement ability, MP passes to neighboring cells (3a) and “conditions” them for more effective viral infection (3b). The synthesized replicase transcribes viral genomic RNA and produces subgenomic RNAs (4). Further MP synthesis is mainly a result of I_2_ sgRNA translation (5).

All the above-mentioned considerations of the mechanisms of early synthesis of MP are based on its synthesis from a dicistronic intermediate length RNA-2 called sgRNA I_2_ (Bruening et al., 1976; Higgins et al., 1976; Beachy and Zaitlin, 1977; Goelet et al., 1982). However, another mechanism for the early synthesis of MP, in addition to subgenomic mRNA, has long been known (Skulachev et al., 1999; Dorokhov et al., 2002; Komarova et al., 2003; Dorokhov et al., 2006; Dorokhov et al., 2017). This mechanism involves the direct binding of ribosomes on the 75-nt sequence of the internal ribosome entry site (IRES) as part of the TMV U1 (IRES_MP,75_^U1^) genomic RNA (Skulachev et al., 1999). A similar element called IRES_MP,75_^CR^ has been detected in the RNA of crucifer-infecting tobamovirus (crTMV) (Dorokhov et al., 1993; Dorokhov et al., 1994; Skulachev et al., 1999). The important role of IRES was demonstrated in the movement-deficient TMV U1-KK6 mutant lacking IRES_MP,75_^U1^ (Lehto et al., 1990). IRES_MP,75_^CR^ insertion restored the intercellular movement of the obtained variant, called TMV U1-K86 (Zvereva et al., 2004). These experiments indicated the fundamental possibility of MP synthesis not only from subgenomic RNA but also directly from genomic RNA (**Figure 1**). It would seem plausible that direct synthesis of MP from genomic RNA would likely be an even faster way to produce MP early in infection, perhaps working together with the subgenomic RNA to achieve this outcome.

## Does the Plasmodesmal Localization Signal (PLS) Direct MP to Pd Using ER-Mediated Trafficking?

The discovery of a specific sequence that is responsible for MP targeting to Pd and designated the plasmodesmal localization signal (PLS) widened our understanding of MP function (Yuan et al., 2016; Yuan et al., 2017; Yuan et al., 2018; Liu et al., 2020). The TMV MP PLS is the first example of a PLS in plant virus MPs; however, its properties are similar to PLSs previously found in cellular transcription factors KN1(Kim et al., 2005) and Dof (Chen et al., 2013). The TMV MP PLS resides in the N-terminal 50 amino acids (aa) (Yuan et al., 2016). The PLS alone, when fused with CFP, is delivered to Pd, as shown by its localization in the plasma membrane in the plasmolysis test, while the whole MP is localized in the Pd cavity and remains associated with the cell wall after plasmolysis. As the PLS-CFP does not go to the Pd cavity, it cannot completely replace the function of the full MP. In support of other portions of the MP having function to target the Pd, MP lacking the PLS has other amino acids (61 to 80 and from 147 to 170) that help direct it to the Pd but in a less efficient manner and not to the next cells (Yuan et al., 2016; Liu et al., 2020). Analysis of cellular factors involved in interactions with MP PLS revealed its ability to bind plant synaptotagmin A (SYTA) (Yuan et al., 2018). SYTA is localized to the plasma membrane (PM), concentrated around Pd and recognized as a tethering factor of ER–PM contact sites (Uchiyama et al., 2014; Levy et al., 2015; Yuan et al., 2018; Ishikawa et al., 2020; Liu et al., 2020).

The results identifying the PLS and its ability to reach the Pd, possibly through interaction with SYTA, do not yet incorporate the known ER-actin network involvement in the intracellular delivery of MP to Pd. In general, the ability of MP to interact with ER was noted more than 30 years ago, and the first studies on infected leaves showed the ability of MP synthesized in the cell to bind ER membranes so tightly that only high concentrations of NaCl or urea or nonionic detergents could detach it (Reichel and Beachy, 1998). It was concluded that MP behaves as an integral ER membrane protein and that the MP affinity for the ER membrane is largely hydrophobic (Reichel and Beachy, 1998). The first topological model developed after research on recombinant MP synthesized in *E. coli* suggested that TMV MP is an integral membrane protein with the N- and C-termini exposed to the cytoplasm and the opposite short loop to the ER lumen. According to that model, MP contains two *α*-helical transmembrane segments, a trypsin-resistant core domain plus 18 aa at the C-terminus of the monomer rapidly removed by trypsin (Brill et al., 2000; Brill et al., 2004; Fujiki et al., 2006). However, the study of MP synthesized *in planta* (Peiró et al., 2014) did not confirm the above-mentioned model of MP as an integral membrane protein. According to the model proposed by Peiró et al. (2014), the hydrophilic 50-aa PLS does not interact with ER membranes. Thus, by what mechanism or mechanisms does PLS lacking the signatures of ER-interacting protein nevertheless appear in Pd as shown by PLS fusions with fluorescent proteins?

We hypothesize that the Golgi apparatus (GA) and cellular secretion mechanisms are involved in this process. However, opposite results have been reported. Studies with brefeldin A at low concentrations (10 μg/ml) (Tagami and Watanabe, 2007) and inhibition of the COP II transport system using a dominant negative GTPase mutant protein, Sar1, did not prevent sustained intracellular MP spread (Genovés et al., 2010). The recently discovered property of the TMV MP PLS to bind SYTA and the role of this interaction in delivery of the MP to the Pd requires further study. The relationship of the MP with SYTA during endosome recycling also requires further study (Lewis and Lazarowitz, 2010).

## Possible Mechanisms of Cell Conditioning to Facilitate the Intercellular Spread of Infection

The TMV MP belongs to a small group of viral MPs that can increase Pd SEL; however, there is no evidence showing their direct effect on Pd components, leading to Pd dilation (Reagan and Burch-Smith, 2020). Recent data indicate that MPs exert their influence indirectly by interacting with factors that then influence Pd conformation, such as callose (Amsbury et al., 2017) and Pd-associated proteins (PdAPs) (Dorokhov et al., 2019). Unlike many PdAPs, callose has been convincingly shown to negatively regulate the Pd aperture (Levy et al., 2007a; Zavaliev et al., 2011; Wu et al., 2018).

One can imagine that the first synthesized MP molecules modify levels of pre-existing cellular components such as callose, which are known to control Pd gating under various physiological conditions (Crawford and Zambryski, 2001; Stonebloom et al., 2012; Grison et al., 2019) and in response to stressful effects (O’Lexy et al., 2018; Grison et al., 2019).

Studies showing how callose may be modified by other proteins and how TMV infection utilizes and/or affects the expression of these proteins have been recently reviewed (Dorokhov et al., 2019). The callose content at the Pd is largely determined by the activity of β-1,3-glucanase (BGs). In turn, TMV infection affects most BGs at the transcriptional level (Levy et al., 2007b). Therefore, while turnip vein clearing virus did not affect *AtBG_ppap* transcription, the transcription of an AtBG2-encoding gene was enhanced (Zavaliev et al., 2013). The introduction of the tobacco *GLU I* gene encoding BG into the TMV genome led to an increase in the local lesion size, which confirms the role of BG as a callose-hydrolyzing enzyme in the cell-to-cell movement of viruses (Bucher et al., 2001).

It must also be borne in mind that mechanical trauma and damage to the cell wall, which are a prerequisite for the virus to enter cells, causes the immediate release of methanol generated by both pre-existing in the cell wall pectin methylesterase (PME) and newly synthesized one (Dorokhov et al., 2012). Methanol vapors activate methanol-inducible genes (MIGs), including *BG* and *non-cell-autonomous pathway protein* (*NCAPP*), which in turn stimulate intracellular trafficking and create favorable conditions for viral infection (Dorokhov et al., 2018a), especially at the early stages. While the synthesis of BG promotes the removal of callose as a plasmodesmal sphincter, NCAPP is a cellular factor that participates in PME/methanol regulation (Sheshukova et al., 2017) and is indispensable for MP functioning (Lee et al., 2003; Lucas et al., 2009).

In addition to the nonspecific conditioning by the mechanisms involved in the response of a plant to trauma and the specific conditioning of cells neighboring the initially infected cell through actions on structures within or proteins that affect the Pd, the MP may influence Pd more indirectly by modulating host protein synthesis. Since MP was shown to have RNA-binding properties (Citovsky et al., 1990) and to inhibit its own mRNA translation *in vitro* (Karpova et al., 1997; Karpova et al., 1999), we suggest that MP may interact with both viral and cellular mRNAs translated on ER-linked polyribosomes and participate in the inhibition of translation of messengers encoding proteins involved in the stress response and regulation of plasmodesmal permeability (Dorokhov et al., 2019; Ganusova and Burch-Smith, 2019).

**Figure 2:**
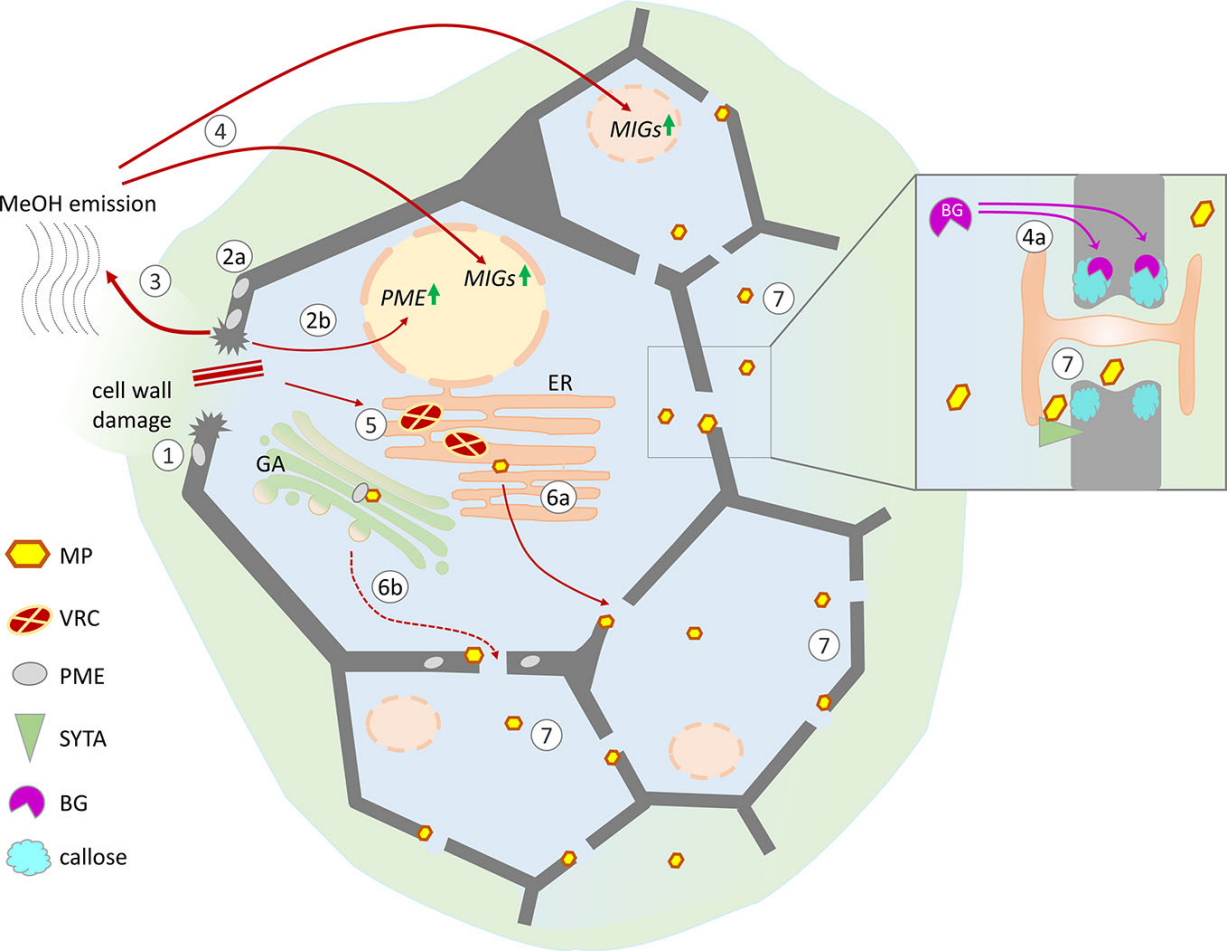
Possible mechanisms of cell conditioning by nonviral factors induced by cell wall trauma and the specific effects of virus-directed MP, leading to the creation of favorable conditions for the intercellular spread of infection. Cell wall damage (1) results in the activation of pre-existing PME (2a) as well as *PME* expression (2b), leading to the increased release of gaseous methanol (3). Methanol-induced genes (MIGs), including β-1,3-glucanase (BG) and NCAPP, are stimulated by methanol (4). MIG induction activates intercellular transport increasing the Pd SEL: BG degrades callose around Pd (4a), and NCAPP is believed to be indispensable for MP functioning. TMV penetrates into the cell through damage to the cell wall, starts to replicate, and forms viral replication complexes (VRCs) on ER membranes (5). MP translated from genomic and subgenomic vRNAs is delivered to the Pd in a tight connection with ER membranes (6a) and possibly *via* the Golgi apparatus in a PME-mediated manner (6b). As MP colocalizes with SYTA in the VRC and Pd, the mechanisms of SYTA transport to the Pd may influence MP delivery. MP may participate in the displacement of negative regulators from the Pd structure and the activation of positive regulators (*e.g.*, SYTA). (7) Moving independently of the genomic viral RNA to the neighboring cells MP increases Pd SEL and creates favorable conditions for the intercellular spread of infection.

Thus, against the background of events caused by cell wall damage, MP might affect Pd gating by interfering with callose metabolism and PdAPs-mediated modification of the host Pd machinery. **Figure 2** summarizes the nonviral factors induced by cell wall trauma and the specific effects of virus-directed MP, leading to the creation of favorable conditions for the intercellular spread of infection.

## Conclusion

To summarize what is known about the TMV MP, this protein is capable of

- forming a stable vRNP complex binding single-stranded RNA in a sequence nonspecific manner;
- targeting to and docking at Pd through the plasmodesmal localization signal;
- self-movement and increasing Pd permeability by influencing the host Pd machinery possibly by interacting with specific cellular components that affect PdAPs or mRNA translation;
- performing Pd gating at the leading edge of the virus infection focus.

Thus, both in a primary infected cell and in neighboring healthy cells, MP may act as a specific “conditioner”, since such preliminary activity is possible only in the cells of a host plant susceptible to TMV. Specific conditioning mechanisms may include MP interaction with BG, NCAPP, mRNA or other factors to open Pd in advance of vRNA.

## Data Availability Statement

The original contributions presented in the study are included in the article/supplementary material; further inquiries can be directed to the corresponding author.

## Author Contributions

ES, YD, TK, KK, and NE analyzed the data, drafted the outline of the manuscript, and wrote the manuscript. ES, YD, and TK revised and finalized the manuscript. All authors contributed to the article and approved the submitted version.

## Funding

The work of YD, TK, and KK was funded by the Russian Science Foundation (project No. 19-74-20031); the work of ES and NE was supported by the Russian Foundation for Basic Research (project No. 18-34-00576).

## Conflict of Interest

The authors declare that the research was conducted in the absence of any commercial or financial relationships that could be construed as a potential conflict of interest.
